# Hers and his: Silk glands used in egg sac construction by female spiders potentially repurposed by a ‘modern’ male spider

**DOI:** 10.1038/s41598-020-63521-7

**Published:** 2020-04-20

**Authors:** Mark A. Townley, Danilo Harms

**Affiliations:** 1 University Instrumentation Center, University of New Hampshire, 23 Academic Way, Durham, NH 03824 USA; 2 Zoological Museum, Center of Natural History, Universität Hamburg, Martin-Luther-King-Platz 3, D-20146 Hamburg, Germany

**Keywords:** Evolution, Structural biology, Zoology

## Abstract

Cylindrical silk gland (CY) spigots distinguish a large clade of modern spiders, the CY spigot clade, which includes all entelegyne spiders and their closest relatives. Following a widespread paradigm, CYs and their spigots are only known to occur in female spiders and they produce silk used in the construction of egg sacs. Here we report the occurrence of a CY spigot or CY nubbin on each posterior median spinneret (PMS) in males (5^th^ stadium and later) of the spider *Australomimetus maculosus*. Late juvenile males had a CY spigot on each PMS, whereas adult males either had a CY spigot or, more often, a non-functional CY nubbin. This indicates that potential CY use by males is at least largely limited to late juvenile instars and is not involved with egg sac construction. Despite the presence of CY spigots in both sexes, sexual dimorphism with respect to CYs was still evident since males lacked the CY spigot on each posterior lateral spinneret present in late juvenile and adult females, and CY spigots of males never had the wide shaft and opening of adult females. This study adds to our knowledge of spinning apparatus variability in modern spiders and demonstrates an exception to the paradigm that, in the CY spigot clade, such spigots are restricted to female spiders.

## Introduction

Recent, extensive phylogenetic analyses of spiders have recognized a clade that includes all “modern” (araneomorph) spiders except the Synspermiata, Filistatidae, and Hypochilidae^1–3^. A synapomorphy of this “CY spigot clade”^2,4^ is the possession of spinneret spigots that are outlets for a type of silk gland known for many years as either tubuliform or cylindrical silk glands (CYs)^5,6^. CY spigots generally occur on both the posterior median spinnerets (PMSs) and the posterior lateral spinnerets (PLSs)^7–9^. Silk drawn from them is used primarily, if not solely, in the construction of egg sacs and so far CYs are only known to occur in females^7–13^. Indeed, this restriction to females can often be used to help distinguish CYs and their spigots from other silk gland types that produce silks for other functions^14–16^. During development in some members of the CY spigot clade, including pirate spiders (family Mimetidae)^17–19^, CY spigots make their first appearance in juvenile females^9,20–27^ though dissections and histological observations have indicated it is not until CY luminal contents are amassed in adults, synchronized to yolk accumulation in the eggs, that CY silk is drawn^7,8,28^.

**Figure 1:**
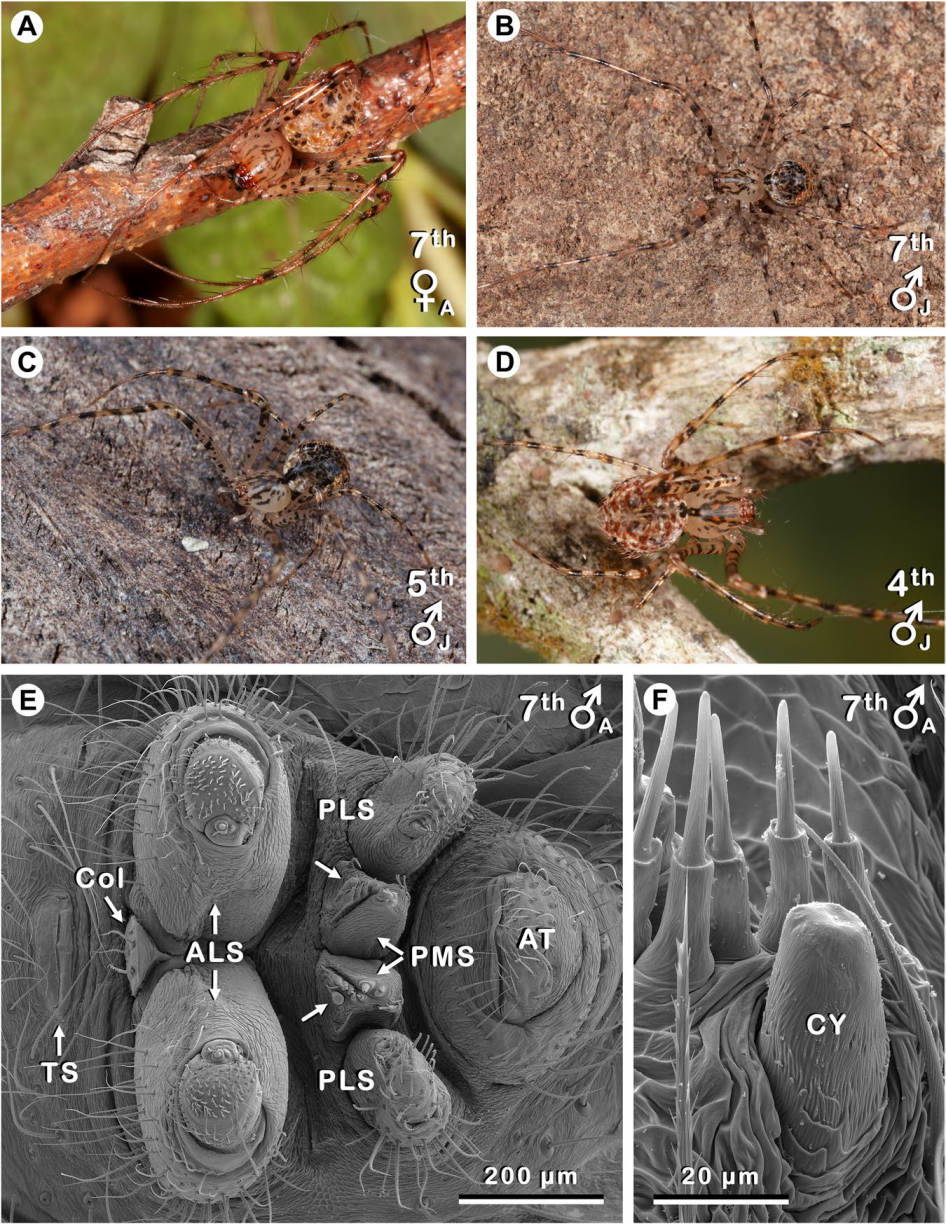
The pirate spider *Australomimetus maculosus* and an overview of its spinnerets. Instar number, sex, and maturity [juvenile (J) or adult (A)] given in upper or lower right of each image. **(A)** Specimen ZMH-A0002038 (see Supplementary Appendix S1), Mt. Colah, New South Wales; spinnerets of this specimen shown in Fig. 2C. **(B)** Specimen ZMH-A0002037, Launceston, Tasmania; note swollen palps on this penultimate male; spinnerets of this specimen shown in Figs. 2B,E, 3C. **(C)** Specimen ZMH-A0002035, Jesmond, NSW; PMS of this specimen shown in Fig. 3A. **(D)** Specimen ZMH-A0002048, Julatten, Queensland. **(E)** Complete set of spinnerets from an adult male. Unlabeled arrows point to CY nubbins on the PMS. Anterior at left. **(F)** Portion of the right PMS from same specimen (image flipped), showing CY nubbin (CY) from lateral perspective. Five unlabeled spigots closest to nubbin are AC spigots. Anterior at right. AT, anal tubercle; Col, colulus; TS, tracheal spiracle. Photos **(A–D)** taken by Greg J Anderson.

Here, we document the consistent occurrence of CY spigots or their nubbins (i.e., vestiges of CY spigots) on PMSs in males (late juvenile and adult) of *Australomimetus maculosu*s (Rainbow, 1904) (Fig. 1A–D); a widespread mimetid spider in forest habitats from eastern Australia that preys extensively on other spiders^29–31^. To our knowledge, neither CY spigots nor CY nubbins have previously been observed in males of a ‘CY spigot clade’ spider. Furthermore, *A. maculosus* seems to be unique in this regard among *Australomimetus* Heimer, 1986 species: our observations were made during an extensive survey of spinning field structures in Australasian pirate spiders^18,19^, when more than 30 species and 300 mimetid specimens (including close relatives of *A. maculosus*) were examined to generate an overview of spinning apparatus ontogeny and variability in spiders that have secondarily given up the web. In none of these other species did males have CY spigots or CY nubbins. Note that CY spigots/nubbins of male *A. maculosus* were restricted to PMSs: they were never observed on PLSs. Note also that we are not suggesting that male *A. maculosus* assist with egg sac construction. Indeed, observations presented below argue against such a role for CYs in these males.

To provide comparisons to previously examined *Australomimetus*^17,19,30,31^, aspects of the spinning apparatus of *A. maculosus* beyond the CY spigots/nubbins of males are also briefly described below.

## Results and Discussion

### Spinning apparatus overview and molts to maturity

**Figure 2:**
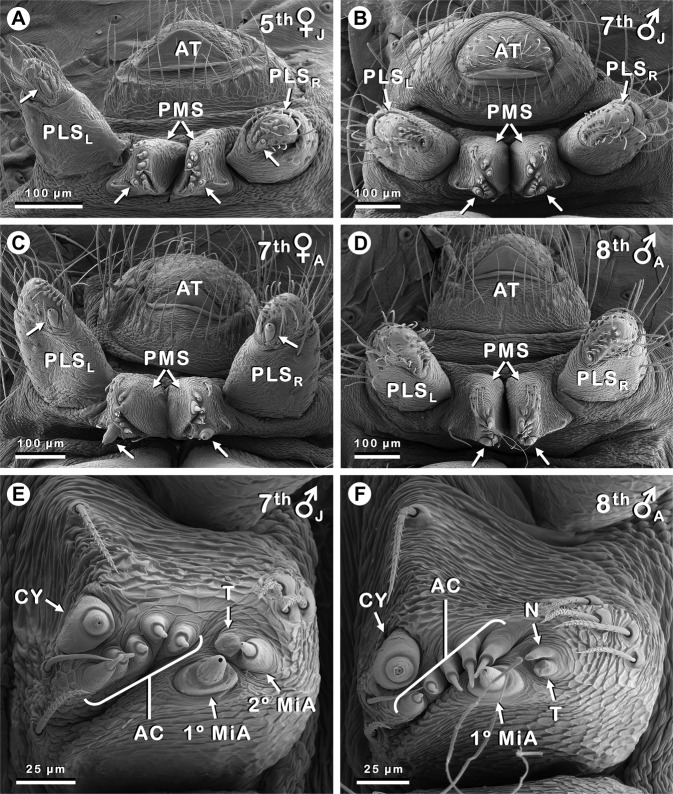
Posterior spinnerets (PLS, PMS) of *Australomimetus maculosus*. Instar number, sex, and maturity [juvenile (J) or adult (A)] given in upper right of each image. Unlabeled arrows in **(A–D)** indicate CY spigots. Females, late juvenile **(A)** and adult **(C)**, have one CY spigot per PMS and PLS. Late juvenile males **(B)** have one CY spigot per PMS only. Adult males may also have one CY spigot per PMS **(D)**, but more often, one or both of these are represented by CY nubbins (see Fig. 1E,F). **(E)** Right PMS of same specimen shown in **(B)** at higher magnification (image flipped). **(F)** Left PMS of same specimen shown in **(D)** at higher magnification. Specimen shown in **(B,E)** also shown in Figs. 1B, 3C. Specimen shown in **(C)** also shown in Fig. 1A. Specimen shown in **(D,F)** also shown in Fig. 3D. **(A–D)** Anterior at bottom; **(E,F)** anterior at left. 1° MiA, 1° MiA spigot; 2° MiA, 2° MiA spigot; AC, AC spigots; AT, anal tubercle; CY, CY spigot; N, 2° MiA nubbin; PLS_L_, left PLS; PLS_R_, right PLS; T, 2° MiA tartipore.

**Figure 3:**
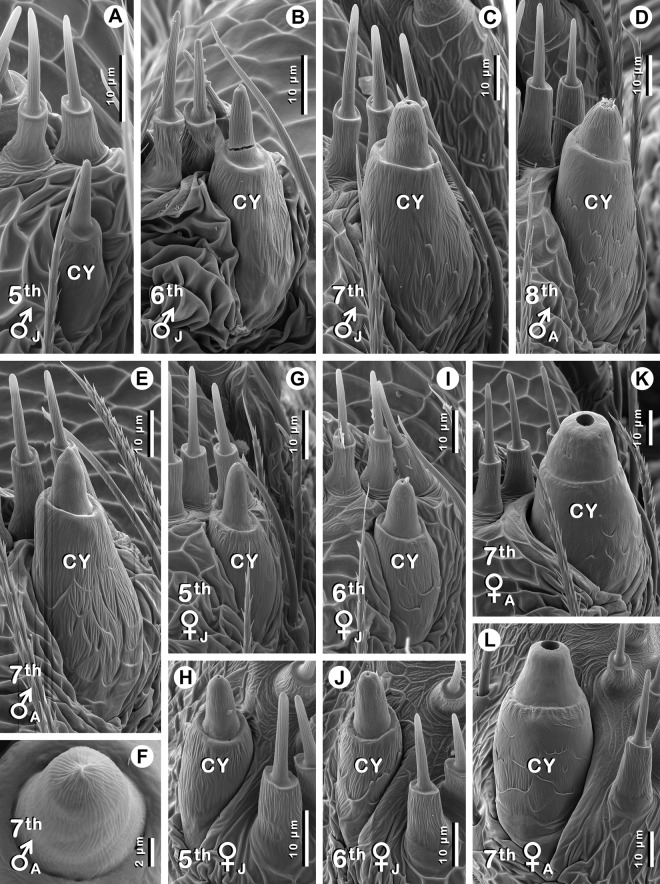
CY spigots (CY) in male **(A–F)** and female **(G–L)** *Australomimetus maculosus*. Instar number, sex, and maturity [juvenile (J) or adult (A)] given in lower left corner of each image. Male CY spigots are on PMS only. Female CY spigots are on PMS **(G,I,K)** and PLS **(H,J,L)**. **(F)** CY spigot shaft from **(E)** at higher magnification and distal perspective: no opening apparent. Unidentified spigots near CY spigots are AC spigots. Specimen shown in **(A)** also shown in Fig. 1C. Specimen shown in **(C)** also shown in Figs. 1B, 2B,E. Specimen shown in **(D)** also shown in Fig. 2D,F. **(G,H)** from same specimen. **(I,J)** from same specimen. **(A–C, E,F)** left PMS; **(D,G,I,K)** right PMS (images flipped); **(J)** left PLS; **(H,L)** right PLS (images flipped). **(A–G,I,K)** Anterior at right; **(H,J,L)** anterior at left.

Overall, the spinnerets of *A. maculosus* (Fig. 1E) resembled those of related species^19,30,31^, but they were unique among members of the CY spigot clade^2,7–16^ due to the presence of a clearly visible CY spigot (Figs. 2B,D–F, 3A–F) or CY nubbin (Fig. 1E,F) on each PMS in all examined males in the 5^th^ stadium or later (*n* = 9). PLSs of these males lacked any CY structures, which is standard and remains without exception among male spiders of the CY spigot clade, but differs from the general pattern of CY spigot occurrence on both PMSs and PLSs in female spiders^7–9^. An inventory of those spinning structures that vary within the Mimetidae^16–19,31–34^ is presented for the spinnerets of *A. maculosus* in Table 1. Additional structures, thus far invariant among mimetids, included the following: In juveniles, each anterior lateral spinneret (ALS) contained one primary (1°) major ampullate silk gland (MaA) spigot^17,19,35,36^, one secondary (2°) MaA spigot, and one 2° MaA tartipore, with a primordium of this tartipore presumably present in 1^st^ instars^17,19,36^ (none examined). Adults of both sexes differed from juveniles in possessing only a nonfunctional vestige of the 2° MaA spigot, called a 2° MaA nubbin. 1° and 2° minor ampullate silk gland (MiA) structures on PMSs, including replacement of juvenile 2° MiA spigots by 2° MiA nubbins in adults (cf. Fig. 2E,F), matched their MaA counterparts exactly.

**Table 1:** Selected spigot, tartipore, and nubbin complements on spinnerets of *Australomimetus maculosus*.

Instar	Sex	*n*	ALS		PMS						PLS		
			PI spigots	PI tartipores	2° MiA spigot	2° MiA nubbin	2° MiA tartipore	AC spigots	CY spigot	CY nubbin	AC spigots	AC tartipores	CY spigot
4th	♂	**2**	18.3 ± 0.75 (17–20)	10.5 ± 0.50 (9–12)	1	0	1 (PM)	3	0	0	7	0	0
5th	♀	**2**	32.3 ± 0.75 (29–34)	18.0 ± 1.00 (17–19)	1	0	1 (AL)	3.8 ± 0.25 (3–4)	1	0	8.3 ± 0.25 (8–9)	0	1
5th	♂	**1**	26.5 (26–27)	13.5 (13–14)	1	0	1 (AL)	3.5 (3–4)	1	0	8.5 (8–9)	0	0
6th Juvenile	♀	**1**	48.0 (46–50)	31.5 (30–33)	1	0	1 (PM)	4.5 (4–5)	1	0	11.5 (11–12)	0	1
6th Juvenile	♂	**1**	50	29.5 (29–30)	1	0	1 (PM)	4	1	0	9.5 (9–10)	0	0
6th Adult	♀	**1**	58.5 (57–60)	29.5 (29–30)	0	1	1 (PM)	4	1	0	11.5 (11–12)	0	1
6th Adult	♂	**1**	67.0 (66–68)	36.0 (35–37)	0	1	1 (PM)	4	0	1	10.5 (10–11)	0	0
7th Juvenile	♂	**1**	67.5 (66–69)	54	1	0	1 (AL)	5	1	0	13	0	0
7th Adult	♀	**4**	66.1 ± 3.60 (59–76)	42.4 ± 2.12 (38–48)	0	1	1 (AL)	4.6 ± 0.31 (4–6)	1	0	14.0 ± 0.79 (12–16)	0	1
7th Adult	♂	**3**	70.0 ± 5.84 (58–79)	44.5 ± 5.92 (34–55)	0	1	1 (AL)	5	0.2 ± 0.17 (0–1)	0.8 ± 0.17 (0–1)	12.5 ± 0.58 (11–14)	0	0
8th Adult*	♂	**2**	75.3 ± 5.25 (70–84)	52.8 ± 8.75 (41–63)	0	1	1 (M)	4.0 ± 1.00 (3–5)	0.5 ± 0.50 (0–1)	0.5 ± 0.50 (0–1)	14.8 ± 0.75 (14–16)	0	0

Numbers of spigots, tartipores, and nubbins are per spinneret; multiply by two for number per spider. If no variation was observed, data presented as integers. Otherwise, data presented as means ± their standard errors (when ***n*** ≥ 2) (calculated using the means from each pair of spinnerets except as noted below*) and, in parentheses, ranges (across all individual spinnerets). ***n*** = number of sets of spinnerets (6 spinnerets/set) examined. See ‘Spinning apparatus overview and molts to maturity’ for corresponding data for 1° and 2° MaA spigots, 2° MaA tartipores, 2° MaA nubbins, and 1° MiA spigots. 2° MiA tartipore positioned anterolateral (AL), medial (M), or posteromedial (PM) to 2° MiA spigot (juveniles) or 2° MiA nubbin (adults). Though no AC tartipores have been observed in *A. maculosus*, we include a column for PLS AC tartipores to acknowledge their presence in some species of *Australomimetus*^19^ (see also Supplementary Fig. S1G,H,S,U). AC tartipores on PMS, on the other hand, have not been observed in any species of *Australomimetus*, nor, to our knowledge, in any araneoid spider. *One of these specimens exhibited developmental abnormalities including a supernumerary right PLS and aberrant left PLS and left PMS. Data from these three spinnerets are not included here. This teratological specimen will be described in detail elsewhere. AC, aciniform silk gland; ALS, anterior lateral spinneret; CY, cylindrical silk gland; 2°MiA, secondary minor ampullate silk gland; PI, piriform silk gland; PLS, posterior lateral spinneret; PMS, posterior median spinneret.

In some adult male mimetid species, the morphology of two piriform silk gland (PI) spigots on each ALS is conspicuously different from that of all other PI spigots^16–18,33,34^; hence, their designation as ‘modified PI (MoPI) spigots’. In other adult male mimetids, including some *Australomimetus* species^18^, careful inspection likewise reveals a morphologically distinct pair of PI spigots, but these do not differ so obviously from other PI spigots and have been called ‘subtle MoPI spigots’^17^. Neither MoPI nor subtle MoPI spigots were observed on the six adult male *A. maculosus* examined here.

Stadium estimates based on spigot/tartipore numbers and 2° MiA tartipore position (see Methods) indicated variation in the number of molts to maturity in *A. maculosus*. Additional observations may expand the range of stadia that potentially include adults, but at a minimum, females may reach adulthood after six or seven molts, males after six, seven, or eight molts (Table 1). Variation in the number of molts to maturity has been observed in a wide range of araneomorph spiders^22,26,37–40^, including other pirate spiders (*Mimetus*^17^, *Australomimetus*^19^), while in other taxa constancy has been reported in one or both sexes^37–39,41^. Both genetic and environmental factors may be responsible for variation^37,38,42–44^.

### CY spigot occurrence

The earliest females of *A. maculosus* examined, two 5^th^ instars, had the full complement of four CY spigots typical for most pirate spiders (except for the genus *Gelanor* Thorell, 1869^32,33^): 1 per PMS and 1 per PLS (Fig. 2A), as did all later females examined, both juvenile and adult (Fig. 2C; Table 1). All these CY spigots were fully formed, consisting of a base and shaft (Fig. 3G–L), but only in adult females (Fig. 1A) did CY spigots have the distinctive morphology that is typical for these taxa: enlarged and rotund with wide-aperture, dome-shaped shafts^32^ (Fig. 3K,L). Thus, CY spigots of juvenile females were narrower than those of adult females, absolutely as well as relative to adjacent aciniform silk gland (AC) spigots. Moreover, differences in diameters of CY spigot openings, through which silk is drawn, were considerable: diameters ranged from 0.3–0.4 µm (5^th^ instars, *n* = 2) and 0.5–0.8 µm (6^th^ instar, *n* = 1) in juvenile females, while the range in adult females was 4.1–4.9 µm (6^th^ instar, *n* = 1) and 3.8–5.4 µm (7^th^ instars, *n* = 4) (Fig. 3G–L). For comparison, openings on AC and 1° MiA spigots across all 5^th^−8^th^ stadia specimens in Table 1 (both sexes) ranged from 0.1–0.4 µm and from 0.9–2.1 µm, respectively (*n* = 17); in 5^th^−7^th^ stadia juveniles, openings on 2° MiA spigots ranged from 0.5–1.0 µm (*n* = 6). CY spigot shafts in juvenile and adult females had finely striated sculpturing but lacked deep longitudinal grooves that can be observed in many mimetids, especially those of the Northern Hemisphere^16–18,32,45^ (Fig. 3G–L).

The earliest males examined, two 4^th^ instars (Fig. 1D), were without CY spigots. However, all nine later males (5^th^ stadium and beyond) had either one CY spigot (Fig. 2B,D) or one CY nubbin (Fig. 1E) per PMS, matching the locations of CY spigots on female PMSs (Fig. 2A,C), though none on the PLS (Table 1). These nine males were from seven localities in New South Wales or Tasmania, with about 1100 km between the most widely separated collection sites (Supplementary Appendix S1). Three of these were juveniles (one each: 5^th^, 6^th^, 7^th^ instars; Fig. 1B,C) and they all had a fully formed CY spigot on each PMS (Fig. 2B,E) with a clearly discernible base and shaft (Fig. 3A–C). Openings on these CY spigots were comparable to those of juvenile females, with diameters of 0.3 µm (5^th^ instar), 0.4 µm (6^th^ instar), and 0.8 µm (7^th^ instar). In contrast, four of six adult males had a CY nubbin only, essentially a base without a shaft (Fig. 1F), on each PMS, and these included one 6^th^ instar, two 7^th^ instars (Fig. 1E), and one 8^th^ instar (Table 1). A third adult male 7^th^ instar had a CY nubbin on the right PMS and a CY spigot on the left PMS (Fig. 3E), albeit without an obvious opening (Fig. 3F); possibly an artifact of storage and/or preparation conditions. Openings were present on spigots of all other silk gland types in this specimen and our observations in this and other *Australomimetus* species have indicated that CY spigot shafts are especially susceptible to degradation (e.g. by enzyme cleaning) and distortion. Indeed, in the one adult male (of six), an 8^th^ instar, that had a complete CY spigot on each PMS (Figs. 2D,F, 3D, Table 1), partial degradation was evident near the openings of these spigots and this was almost certainly an artifact, not shared by other spigots.

There was a trend for CY spigot shafts to become stouter (decreased height/width) from one stadium to the next (cf. Fig. 3A–C). In females, this trend ended with a flourish, the final molt producing an especially dramatic transformation to a large and domed shape (cf. Fig. 3I–L). By contrast, in adult males, shafts (if present) retained a basically juvenile morphology, comparable to females before the final molt (cf. Figs. 2F, 3D–J). This included an absence of wide openings: despite some degradation potentially inflating measurements, diameters of 0.9 µm and 1.1 µm were obtained for openings in the one adult male (8^th^ instar) with measurable PMS CY spigot shafts (Figs. 2D,F, 3D), only modestly wider than those of late juvenile females and far from those of adult females (see above).

In summary, CY spigots or their vestiges were present in all male specimens we examined after the 4^th^ stadium, but while three such juveniles displayed an intact CY spigot (base and shaft) on each PMS (Figs. 2B,E, 3A–C), only 25% of the PMSs on six adult males (i.e., 3 of 12 PMSs) were so endowed (Figs. 2D,F, 3D,E); the remaining 75% had a CY nubbin (base only), incapable of acting as silk conduits (Fig. 1E,F). Though not conclusive, CY spigots in males are strong external indicators of internal CYs, and CY nubbins are indicators of fully formed CY spigots earlier in ontogeny.

### CY spigot sexual dimorphisms

The usual sexual dimorphism regarding CYs that exists among members of the CY spigot clade is that females possess CY and their spigots whereas males lack them altogether. In males of *A. maculosus*, CY spigots were clearly observed. Despite this, CY sexual dimorphism was still apparent in the form of CY spigot distribution, morphology, and, possibly, ontogeny. Females within the CY spigot clade usually have CY spigots distributed on both PMSs and PLSs^7–9^. Females of *A. maculosus*, like other *Australomimetus*^17,19,30,31^, were no exception and one CY spigot was present on each of these four spinnerets (Fig. 2A,C). Males instead had one CY spigot/nubbin on each of the PMSs, but none on the PLSs (Figs. 1E, 2B,D).

Adult females of most mimetids (except *Gelanor*) are known for having enlarged and stout CY spigots with dome-shaped shafts displaying wide apertures^16–19,30–32,45^. Again, adult females of *A. maculosus* conformed to this description (Fig. 3K,L) while adult males did not. In some instances, this was because only a CY nubbin formed on an adult male PMS (Fig. 1F). However, even when a complete CY spigot formed, its morphology was essentially like that of a juvenile (male and female), with the shaft lacking a wide aperture (Fig. 3D,E). An interesting parallel to this atypical CY spigot distribution and morphology exists in another mimetid genus, albeit in the opposite sex. In females of some species of the African genus *Anansi* Benavides and Hormiga, 2017, one CY spigot is present on each PMS only and these spigots lack an enlarged, rotund, wide-aperture morphology^34^. Male *Anansi*, like all examined mimetids except for *A. maculosus*, appear to lack CYs^34^.

Less certain is a sexual dimorphism concerning the stadium in which CY spigots first appear in the ontogeny of *A. maculosus*. In this study, because no females earlier than 5^th^ instars were examined, we can only say with confidence that full sets of CY spigots (1/PMS, 1/PLS) were present in juvenile females by the 5^th^ stadium (Fig. 2A, Table 1). However, observations from other species suggest their first appearance likely preceded this stadium. In studies of *Mimetus puritanus* Chamberlin, 1923^17^, *Mimetus notius* Chamberlin, 1923^17^, and *Australomimetus spinosus* Heimer, 1986^19^, CY spigots first appeared in female 3^rd^ instars, though often not the full set of four CY spigots in *A. spinosus*. By the 4^th^ stadium, however, all four CY spigots were invariably present, as they were in the only examined female 4^th^ instar of *Australomimetus djuka* Harms and Harvey, 2009^19^ and in female 4^th^ instars of six other species of *Australomimetus* we have examined. In contrast, assuming our identifications of two 4^th^ stadium *A. maculosus* as males are correct (see Methods), it appears that the PMS CY spigots of males do not make their first appearance until the 5^th^ stadium (Table 1, Fig. 3A). More juveniles will need to be examined to confirm or disprove a sexual dimorphism in CY spigot ontogeny.

### Role of CY in male *A. maculosus*

The older literature contains opposing or indistinct views regarding the presence or absence of CYs in male spiders^46^. Consequently, roles played by apparently non-existent CYs in males were sometimes hypothesized, including contributing fibres to sperm webs^47^ and producing the core fibres of orb web sticky spirals^48^; the latter later shown to be products of flagelliform silk glands^49,50^. The presence of PMS CY spigots in male *A. maculosus* prompts us to consider anew potential CY function in males, if only for this single species at present. Relevant to such a consideration, we emphasize that a majority of CY structures on PMSs of adult male *A. maculosus* were non-functional nubbins (Fig. 1E,F, Table 1) whereas intact and apparently functional CY spigots were invariably present on both PMSs on the admittedly small number (3) of late juvenile males examined (Figs. 2B,E, 3A–C). Moreover, in the minority of instances in which an adult male PMS was equipped with an intact CY spigot, it retained a basically juvenile morphology (Figs. 2D,F, 3D–F), not the rotund, wide-aperture morphology of adult females (Figs. 2C, 3K,L). Thus, if CYs in male *A. maculosus* do play a role, these observations suggest they are largely, if not exclusively, used by late juvenile males. This conclusion argues strongly against male *A. maculosus* assisting in any capacity with egg sac construction.

Certainly, *A maculosus* does not differ drastically in behaviour and morphology from other Australian pirate spiders despite its adaptability to habitats ranging from natural rainforests to urbanized areas, and to an apparently wide prey spectrum that may include comb-footed spiders (family Theridiidae), nursery web spiders (Pisauridae), ecribellate orb weavers (Araneidae), cribellate orb weavers (Uloboridae), daddy-long-leg spiders (Pholcidae), and sheet web builders (Desidae)^29^. Nothing is known about behaviours specific to late juvenile or adult males of this species and the possible prey spectrum is based on host webs in which the spiders have been observed in the field^29^. Behavioural studies and controlled laboratory experiments may clarify the use of CYs by males, revealing behaviours that may involve CY silk production.

### Drawing of CY silk by juveniles

We cannot assume that CY spigots in juvenile females are used in the drawing of CY silk^19^ since CYs exhibit little silk synthesis prior to the onset of vitellogenesis in adults^7,8,21,27,28,51,52^. Likewise, we cannot assume *a priori* that silk is drawn from CY spigots of juvenile male *A. maculosus*, though it does at least seem more probable than in juvenile females. This is because CYs in females perform a definite role in adults, contributing silk fibres to egg sacs, and explanations proposed for CY spigots in juvenile females do not imply use of CYs by these juveniles: namely, that such spigots reflect earlier-maturing ancestors^24^ or act as placeholders for the functioning CY spigots of adult females^23^. In contrast, the non-functional CY nubbins observed in a majority of adult males argues against a significant role for CYs in adult males, making the drawing of silk by late juvenile males, for a yet unknown purpose, the most likely explanation for CY spigot occurrence in males. It will be of interest to determine if late juvenile males differ from late juvenile females of *A. maculosus* in their CY development, especially with respect to quantities of silk dope accumulated in the lumen, and whether morphological or histological changes occur in the CYs following the final molt in males. As noted earlier^19^, if juvenile females do draw CY silk, the fibres are presumably considerably narrower than those drawn by adult females during egg sac construction given differences in diameters of their CY spigot openings (see CY spigot occurrence). For the same reason, any CY silk drawn by males is likewise expected to be much narrower.

### Male CY spigots are unique to *A. maculosus*

As the type species of the genus, *A. maculosus* is in most respects a fitting representative; its somatic and genital morphology is typical^30,31,53^ despite its relatively large body size^30^. Conversely, the possession of PMS CY spigots by males is clearly a unique feature. We have examined spinnerets by SEM from males of 17 other species in this genus (*A. annulipes* Heimer, 1986, *A. audax* (Hickman, 1929), *A. aurioculatus* (Hickman, 1929)^17,31^, *A. catulli* (Heimer, 1989), *A. daviesianus* Heimer, 1986, *A. diabolicus* Harms and Harvey, 2009^17^, *A. djuka* Harms and Harvey, 2009^19^, *A. hartleyensis* Heimer, 1986, *A. hirsutus* Heimer, 1986, *A. japonicus* (Uyemura, 1938), *A. kioloensis* Heimer, 1986, *A. men*dax Harms and Harvey, 2009, *A. mendicus* (O. Pickard-Cambridge, 1880), *A. pseudomaculosus* Heimer, 1986^17^, *A. spinosus* Heimer, 1986^19^, *A. sydneyensis* Heimer, 1986, *A. tasmaniensis* (Hickman, 1929)^17^) (Supplementary Fig. S1A–M if no reference given), as well as spinnerets from males of another eight as yet undescribed species (Supplementary Fig. S1N–U), and in none of these were CY spigots or CY nubbins observed.

Our results mark this species as a unique representative of the CY spigot clade that deviates from a widely held paradigm for modern spiders, but the secrets underlying its unique male morphology have yet to be revealed. We suggest behavioural, anatomical, and developmental studies on this species to explore the basis of this unique arrangement in spider evolution.

## Methods

### Spinneret examination

Spinnerets from 19 specimens of *Australomimetus maculosus* (Table 1), preserved in 75% ethanol, were prepared and examined by scanning electron microscopy (SEM) as described previously^19^. Collection and repository data for these specimens is given in Supplementary Appendix S1. All were collected in eastern Australia, from Far North Queensland to Tasmania. Eleven of the 19 specimens were males from eight localities, though only one male, a juvenile in the 4^th^ stadium (Fig. 1D), was collected in Far North Queensland. The other ten males, including nine that were in the 5^th^ stadium or later (i.e., those with CY spigots or nubbins) (Fig. 1B,C), were collected in New South Wales (five locations) or Tasmania (two locations).

Diameters of spigot openings were obtained from SEM images using the measurement tool in Tescan Lyra3 Control Software. Each spigot aperture was measured twice: at its widest point and perpendicular to this, with the mean then taken.

To facilitate comparisons among spinnerets of the same type (ALS, PMS, or PLS), any single spinneret scans from the right side of the opisthosoma were flipped in Microsoft PowerPoint version 1808 so they appear to be from the left side: actual handedness (right, left) is stated in the figure legends.

### Terminology

Spinneret terminology follows previous research^19^. Briefly, a **nubbin** is a vestigial silk gland spigot while a **tartipore** is a short conduit that forms during proecdysis (the preparatory period before ecdysis) within the developing exoskeleton, surrounding a silk gland duct. This opening or pore allows the duct to remain attached to a spigot on the older, overlying exoskeleton so that silk can still be drawn from the spigot despite the intervening new exoskeleton. After ecdysis, the tartipore, though no longer functional, is still visible in the new exoskeleton.

Note that silk gland abbreviations such as ‘CY’ and ‘AC’ stand for ‘cylindrical silk gland’ and ‘aciniform silk gland’ rather than just ‘cylindrical’ and ‘aciniform’. Thus, a term like ‘CY spigot’ should be understood as meaning ‘cylindrical silk gland spigot’. In figures, to reduce labeling, CY spigots/nubbins are labeled ‘CY’ alone, but the legend states that this label stands for CY spigot or CY nubbin.

No early instars of *A. maculosus* were examined during this study but for instar/stadium assignments to be most meaningful we need to define the 1^st^ instar and 1^st^ stadium, an **instar** being the spider itself and a **stadium** being the period between ecdyses or following the final ecdysis. We follow Downes^54^ in calling the spider that hatches from the egg a **postembryo**. The **1**^**st**^ **instar** emerges and the **1**^**st**^ **stadium** begins after the postembryo has molted and discarded its old exoskeleton. At least among most araneoids, a functioning spinning apparatus is available to 1^st^ instars, but not to postembryos.

### Species, instar/stadium, and sex determinations

Juvenile specimens of *A. maculosus* were distinguished from other *Australomimetus* species on the basis of somatic characters^30^; adult specimens on the basis of characteristic genital structures: prominent, complex palpal bulbs in males, sclerotized epigynum and spermathecae in females^30,53,55^. The presence or absence of these genital structures was unequivocally ascertained and, when present, guaranteed the specimen had attained full maturity and completed its final molt.

To estimate the stadium an individual was in at the time of death, we counted PI spigots, PI tartipores, and AC spigots (Table 1) since these increase in number, albeit with variation, during ontogeny. Stadium estimates were further refined by noting the position of the post-functional 2° MiA tartipore relative to the 2° MiA spigot (if a juvenile) or 2° MiA nubbin (if an adult). If this tartipore was (postero)medial to the 2° MiA spigot/nubbin (see Fig. 2F), the most likely even-numbered stadium was assigned; if anterolateral (see Fig. 2E), the most likely odd-numbered stadium was assigned. This pattern has been observed consistently in seven other species of *Australomimetus* that, like *A. maculosus*, possess 2° MiA, as opposed to others that have lost these silk glands^19^ (See Supplementary Fig. S1A–C,I,O,Q,R for examples of species without 2° MiA). Moreover, the same pattern has previously been observed in two *Mimetus* Hentz, 1832 species^17^ and two araneid species (*Neoscona theisi* (Walckenaer, 1841)^22^, *Araneus cavaticus* (Keyserling, 1881)^17,23^), though *Larinioides cornutus* (Clerck, 1757) may not be consistent (Yu and Coddington^22^ figs. 8–11, note though that their ‘2^nd^ instar’ is a ‘1^st^ instar’ by our definition).

The sex of juveniles was determined by examining palps for distal swelling, indicative of forthcoming male palpal bulbs. Though swelling is most pronounced in penultimate males (Fig. 1B), it can be discerned earlier than this. Indeed, two estimated 4^th^ instars identified as males exhibited a greater degree of palp swelling than was observed in late juveniles and adults that were unquestionably female.

## Supplementary information

Supplementary information.

## Data Availability

An additional figure and an appendix have been uploaded as part of the electronic Supplementary Material. Higher resolution spinneret SEM files are available from M.A.T.
