# Supplementary material for: Hers and his: Silk glands used in egg sac construction by female spiders potentially repurposed by a ‘modern’ male spider
Source: Sci Rep. 2020 Apr 20;10:6663. doi: 10.1038/s41598-020-63521-7 (PMC7170858; doi:10.1038/s41598-020-63521-7)
Supplement: Supplementary file 1 — Supplementary information. [file 41598_2020_63521_MOESM1_ESM.pdf]

Hers and his: Silk glands used in egg sac construction by female spiders potentially repurposed by a ‘modern’ male spider

Mark A. Townley[1,*] **and Danilo Harms**[2]

[1]University Instrumentation Center, University of New Hampshire, 23 Academic Way, Durham, NH 03824, USA.

[2]Zoological Museum, Center of Natural History, Universität Hamburg, Martin-Luther-King-Platz 3, D-20146 Hamburg, Germany.

*corresponding author: mark.townley@unh.edu

Supplementary Material, including:

- Supplementary Figure S1
- Supplementary Appendix S1

Supplementary Figure S1. Posterior spinnerets from males of 19 species of *Australomimetus*, demonstrating the absence of external CY structures in males of these congeners of *A. maculosus* (Fig. 1A-D), a species in which males exhibit a CY spigot or CY nubbin on each PMS (Figs. 1E, F, 2B, D-F, 3A-F). Each row of SEM scans shows one PLS and a PMS pair. Though even male *A. maculosus* lack CY structures on PLSs (normal for male entelegynes), because female *Australomimetus* have a CY spigot on each PLS as well as on each PMS, we present a representative male PLS for each species to rule out all known sites of CY spigots in this genus. Anterior is to the left for all PLS images and at bottom for all PMS images. All spigots on PLSs are AC spigots (AC), as indicated in **(A)**. Unlabeled spigots on PMSs, anterior and/or lateral to the 1° MiA spigot (1° MiA), are also AC spigots. CY structures, if present, would be expected anterolateral to these PMS AC spigots, or anterior to the most proximal AC spigots on PLSs (Figs. 1E, 2). Unlabeled arrows on PLSs in **(G, H, S, U)** point to AC tartipores[19]. Numbers of male specimens examined by SEM for CY structures are given parenthetically below, with collection and repository data for these specimens given in Supplementary Appendix S1. **(A)** *A. annulipes*, adult ♂ (*n* = 1); **(B)** *A. audax*, adult ♂ (*n* = 3); **(C)** *A. catulli*, adult ♂ (*n* = 2); **(D)** *A. daviesianus*, adult ♂ (*n* = 3); **(E)** *A. daviesianus*, antepenultimate ♂ (*n* = 1); **(F)** *A. hartleyensis*, adult ♂ (*n* = 3); **(G)** *A. hirsutus*, adult ♂ (*n* = 1); **(H)** *A. japonicus*, adult ♂ (*n* = 1); **(I)** *A. kioloensis*, adult ♂ (*n* = 1); **(J)** *A. mendax*, adult ♂ (*n* = 6); **(K)** *A. mendax*, penultimate ♂ (*n* = 1); **(L)** *A. mendicus*, adult ♂ (*n* = 1); **(M)** *A. sydneyensis*, adult ♂ (*n* = 3); **(N)** *Australomimetus* sp. 'NZ', adult ♂ (*n* = 2); **(O)** *Australomimetus* sp. 'AU1', adult ♂ (*n* = 1); **(P)** *Australomimetus* sp. 'AU2', adult ♂ (*n* = 1); **(Q)** *Australomimetus* sp. 'AU3', penultimate ♂ (*n* = 1); **(R)** *Australomimetus* sp. 'AU4', adult ♂ (*n* = 1); **(S)** *Australomimetus* sp. 'NB', adult ♂ (*n* = 1); **(T)** *Australomimetus* sp. 'NC', adult ♂ (*n* = 1); **(U)** *Australomimetus* sp. 'PNG', adult ♂ (*n* = 1). **(A, C-F, H-J, M-P, S, T)** left PLS shown; **(B, G, K, L, Q, R, U)** right PLS shown, image flipped horizontally. 2° MiA, 2° MiA spigot; N, 2° MiA nubbin; T, 2° MiA tartipore.

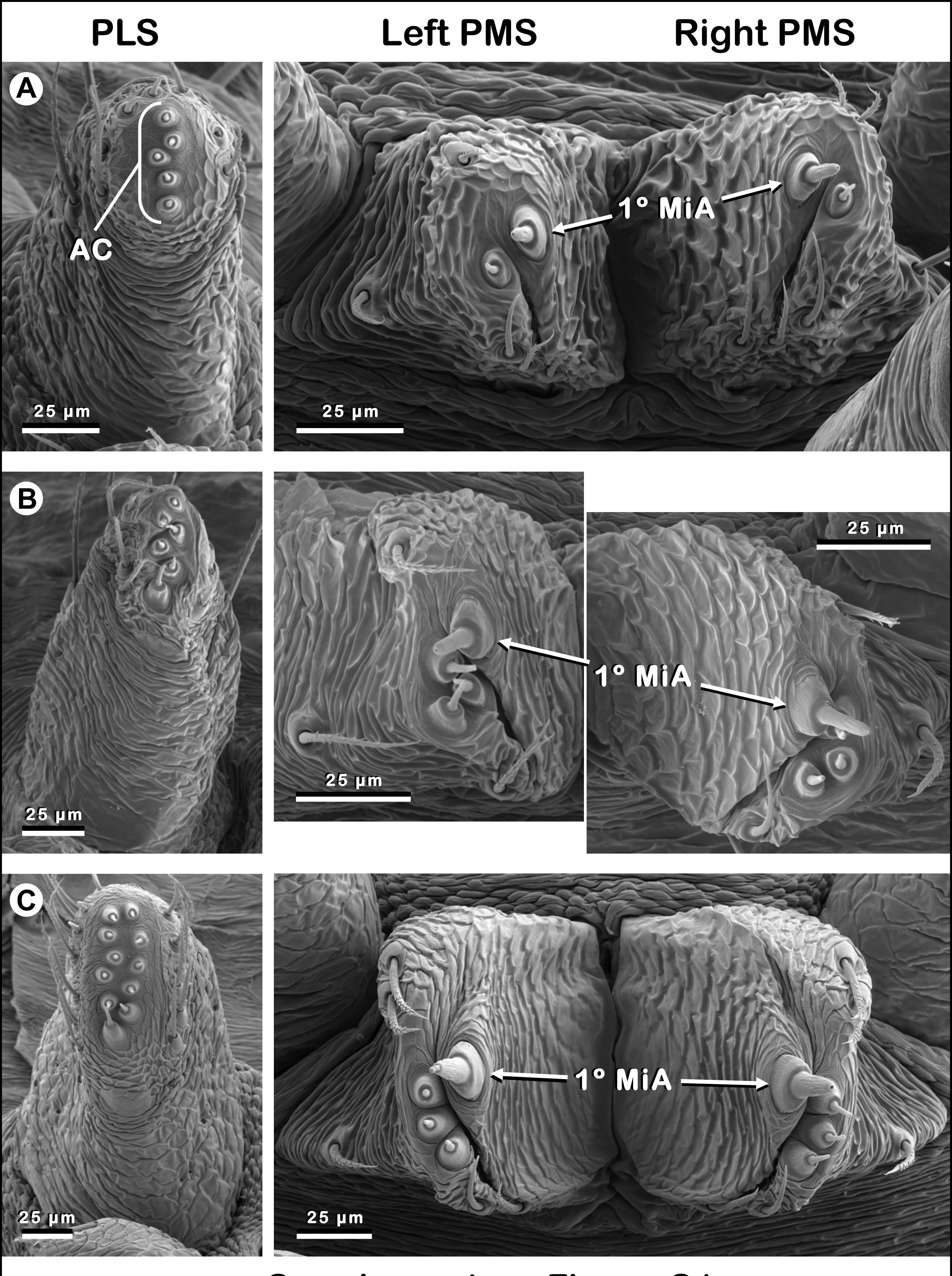

Supplementary Figure S1

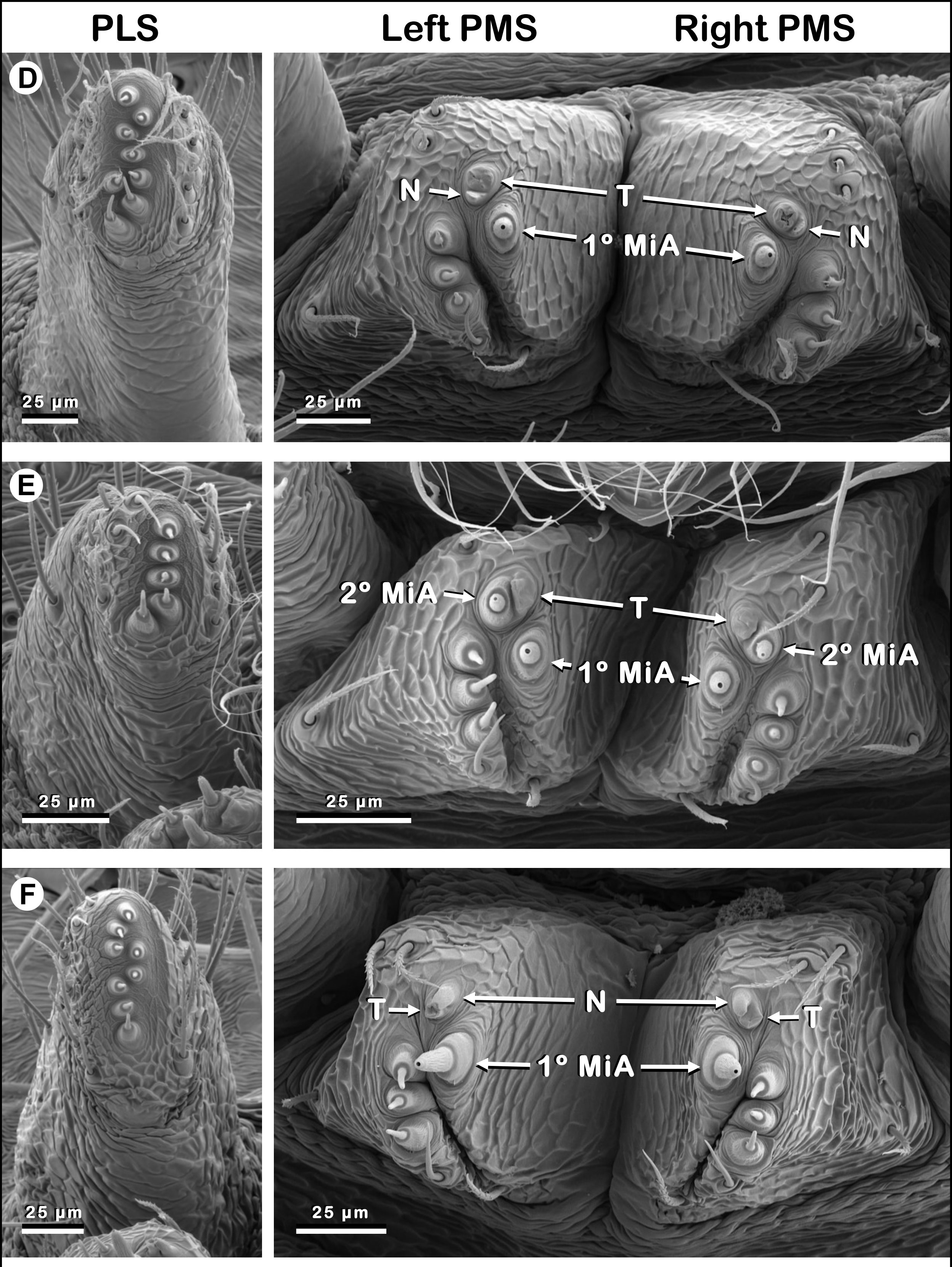

Supplementary Figure S1

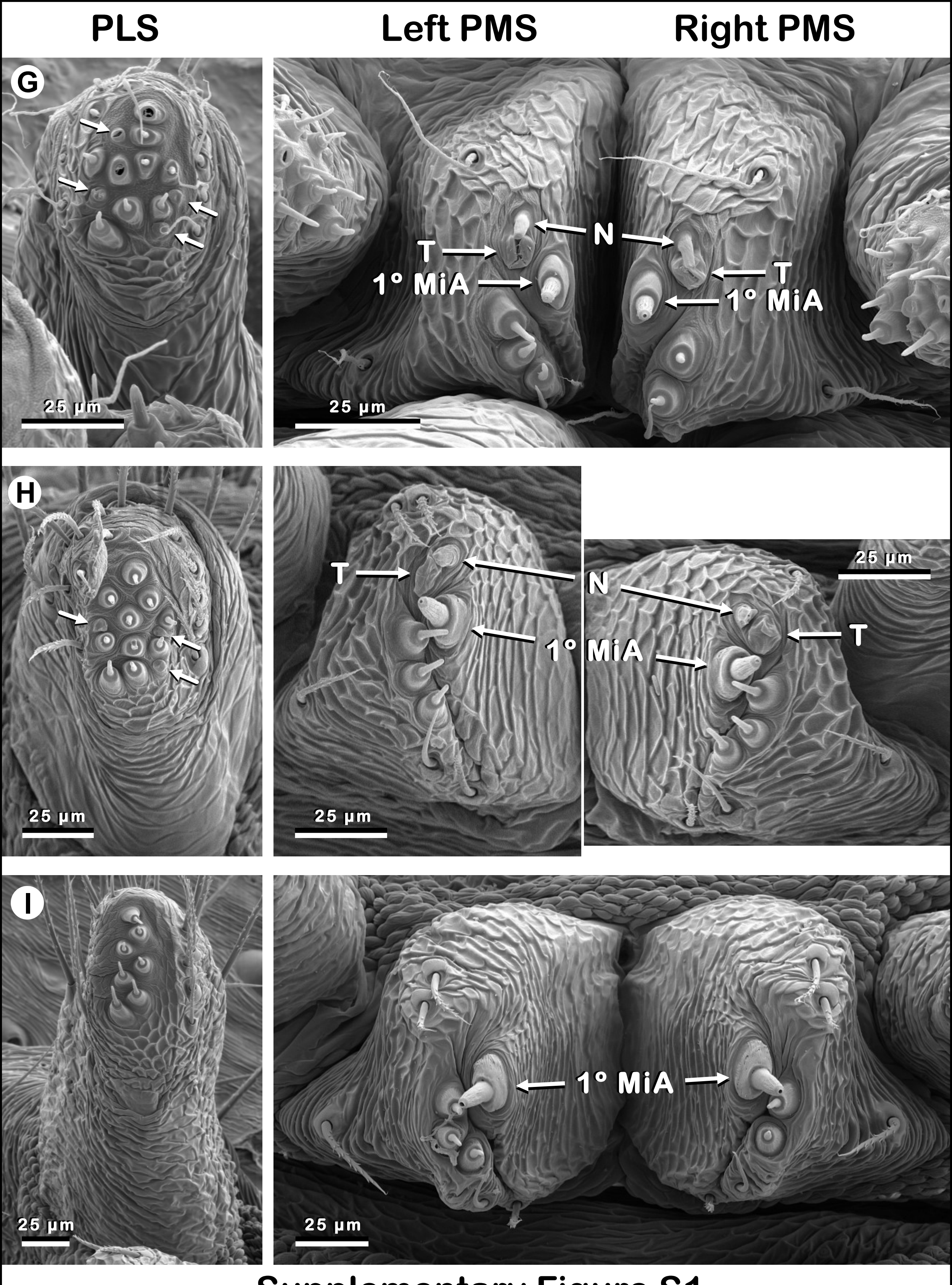

Supplementary Figure S1

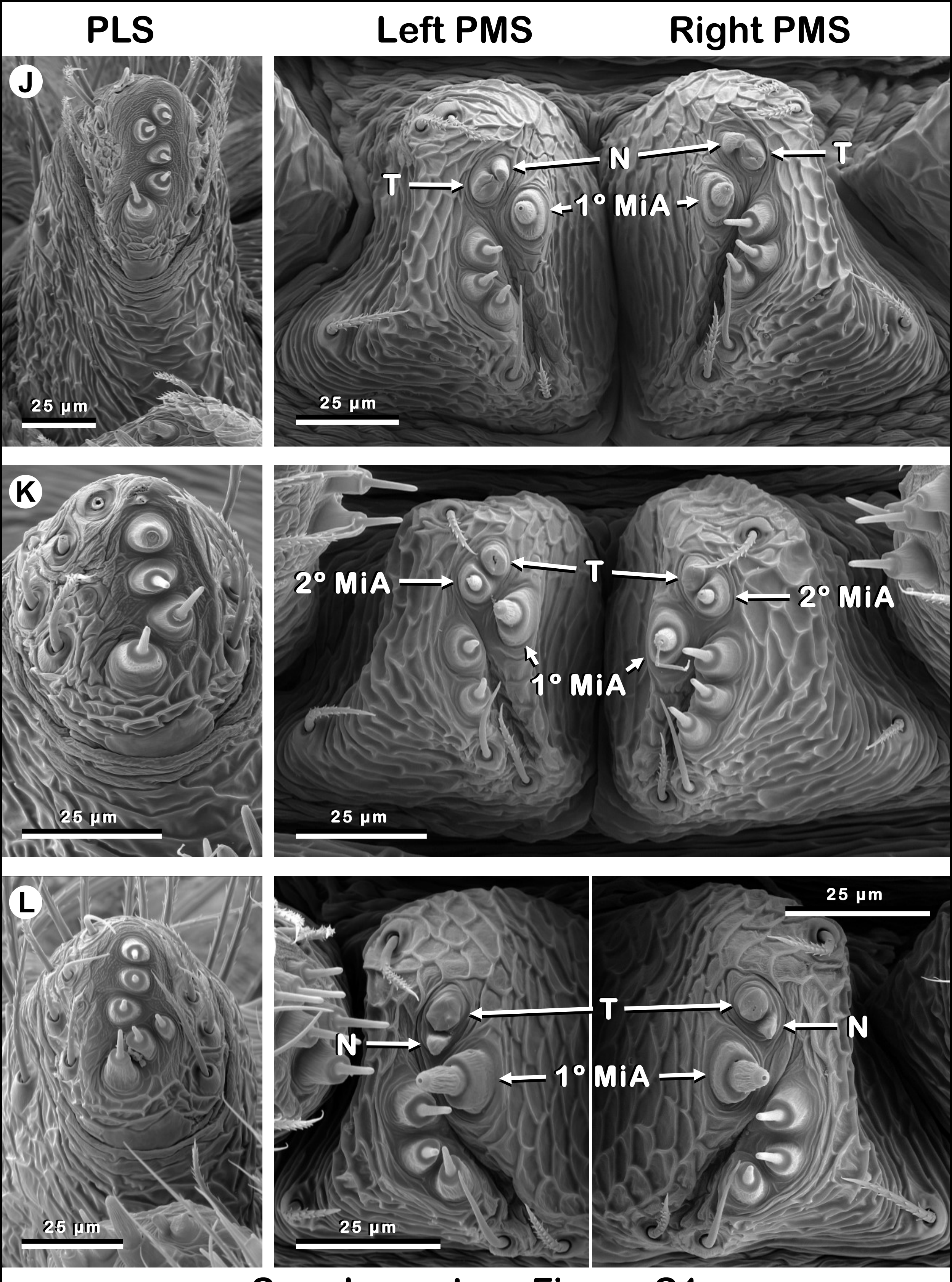

Supplementary Figure S1

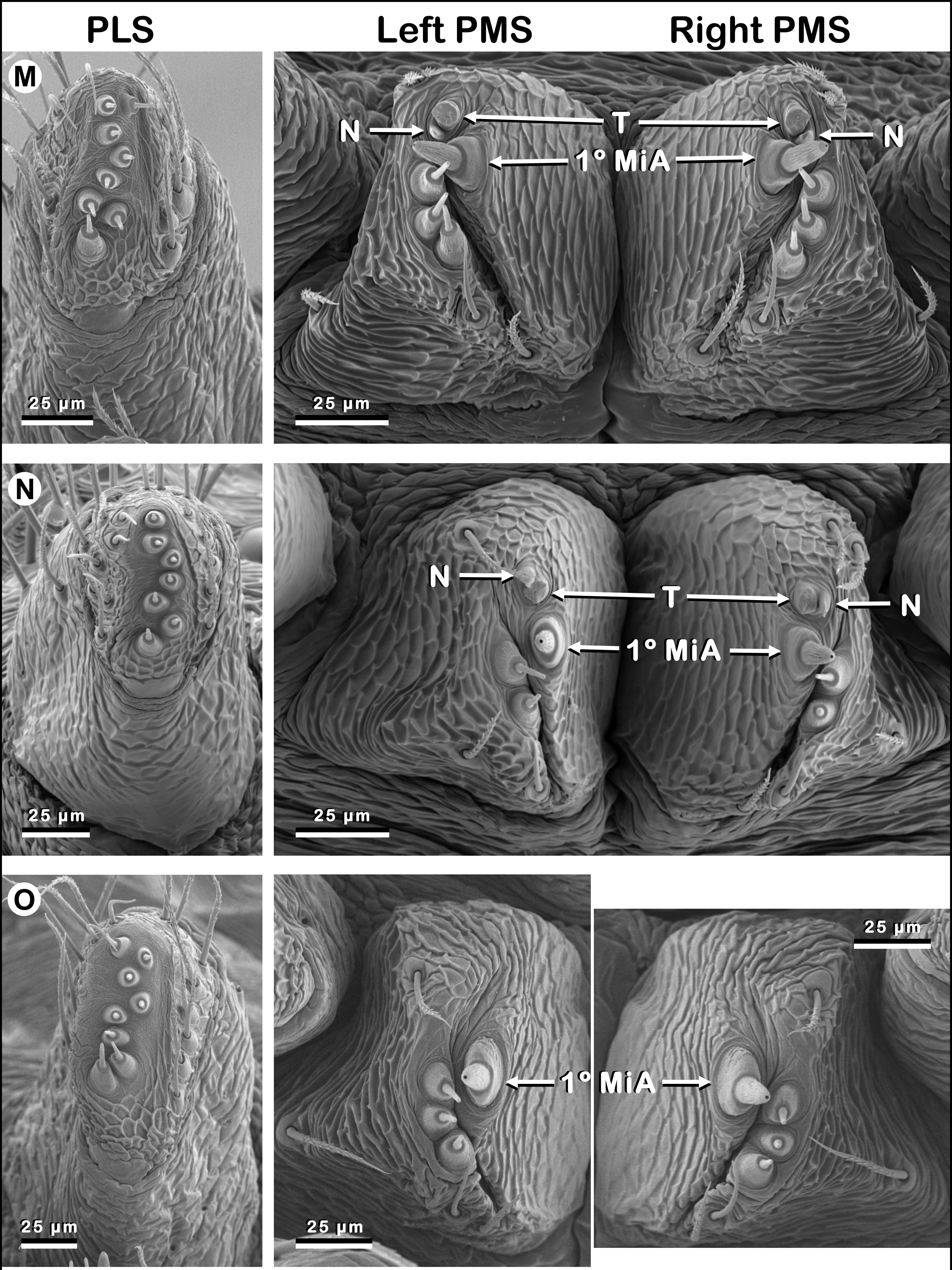

Supplementary Figure S1

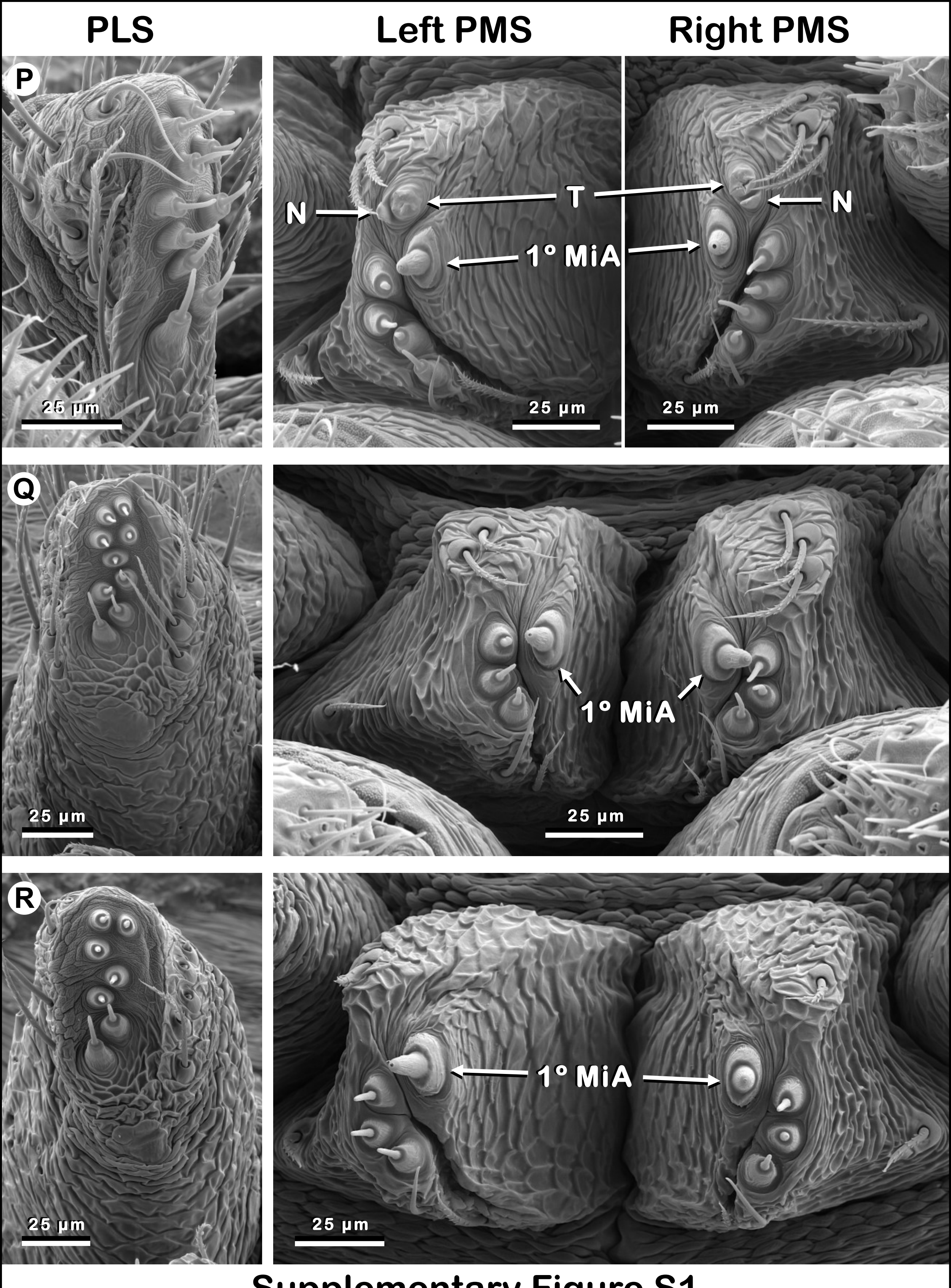

Supplementary Figure S1

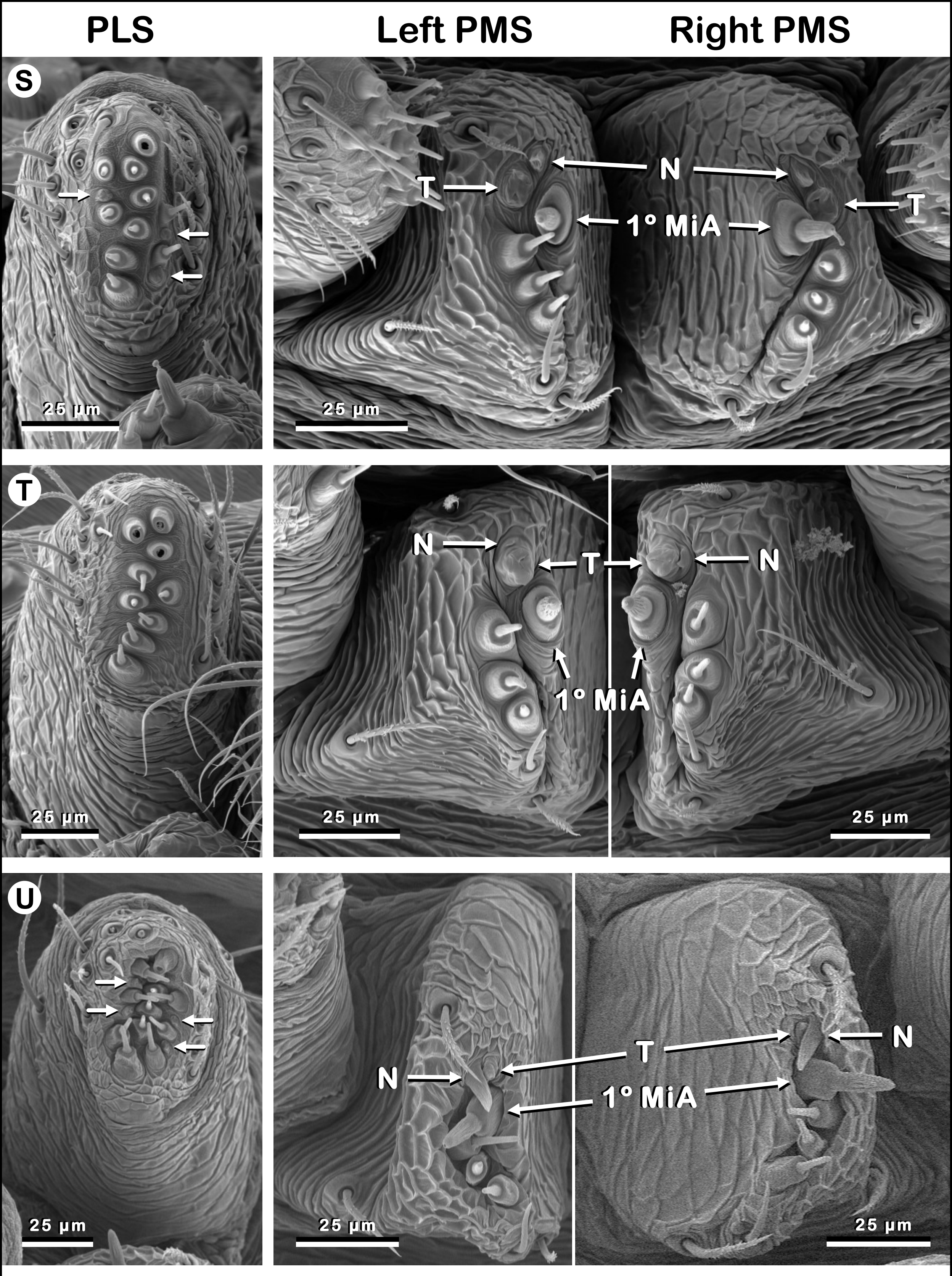

Supplementary Figure S1

Supplementary Appendix S1. Collection and repository data for specimens on which spinnerets were examined by SEM, including those indicated or shown in Supplementary Fig. S1.

Repositories: Australian Museum (AM); Australian National Insect Collection (ANIC); Queensland Museum (QM); Western Australian Museum (WAM); Universität Hamburg - Zoological Museum (ZMH).

Australomimetus annulipes Heimer, 1986. **Australia: New South Wales:** 1♂ Lord Howe Island, Stevens Reserve, fogging tree trunks (greybark, maulwood), 31°31'32"S 159°03'51"E, 25 February 2001, coll. G Milledge (**AM**-KS70600).

Australomimetus audax (Hickman, 1929). **Australia: New South Wales:** 1♂ Bondi State Forest south of Bombala Woodlot 1, 37°08'S 149°09'E, 6 May 1980, det. D Harms, December 2014 (**AM**-KS18174). **Tasmania:** 2♂ bred from egg sacs found at E. Risdon, 42°50'S 147°21'E, 12 October 1967, coll. VV Hickman (**AM**-KS30567).

Australomimetus catulli (Heimer, 1989). **Australia: Queensland:** 2♂ Conondale National Park off Booloumba Creek Road (ArPTC#64), sifting/beating in subtropical rainforest near Booloumba Creek, 26°38'50"S 152°39'05"E (WGS84), 30 April 2010, coll. MG Rix, D Harms (**ZMH**-A0002021).

Australomimetus daviesianus Heimer, 1986. **Australia: Queensland:** 1♂ near Kuranda, Black Mountain Road, Cassowary House, ca. 16°49'S 145°38'E, 29 September 2011, coll. GJ Anderson (**ZMH**-A0002033, GJA6330); 1♂ Black Mountain National Park, r/f patch at base of mtn, ca. 50 m S of lookout, 15°39'05"S 145°13'13"E, 16 May 2000, coll. G Milledge, H Smith (**AM**-KS66451); 1♂, 1 juvenile ♂ (antepenultimate) Thornton Peak N of Daintree, Rainforest Site 40, foliage, 16°10'S 145°22'E, 955 m elev., November 1975, coll. M Gray, det. D Harms December 2014 (**AM**-KS0557).

Australomimetus hartleyensis Heimer, 1986. **Australia: Queensland:** 1♂ near Kuranda, Black Mountain Road, Cassowary House, 16°49'S 145°38'E, 30 December 2010, coll. GJ Anderson (**ZMH**-A0002034, GJA5910); 2♂ N of Bloomfield, Fritz Creek, Rainforest Site 33, foliage, single strands, 15°52'S 145°21'E, 31 December 1975, coll. M Gray, det. D Mott July 1996 (**AM**-KS0455).

Australomimetus hirsutus Heimer, 1986. **Australia: Queensland:** 1♂ NW of Bundaberg, Bulburin (Forestry Nursery), Rainforest Site 3A, foliage, 24°31'S 151°29'E, 580 m elev., March 1975, coll. M Gray, C Horseman, det. D Mott July 1996 (**AM**-KS0098).

Australomimetus japonicus (Uyemura, 1938). **Japan: Kyushu:** 1♂ Fukuoka Prefecture, Sugao Waterfall, 33°47'02"N 130°49'08"E, coll. D Harms, MS Harvey, Y Konishi (**ZMH**-A0002022).

Australomimetus kioloensis Heimer, 1986. **Australia: ACT:** 1♂ 6 km NE of Piccadilly Circus, Wombat Creek, 35°19'S 148°51'E, 750 m elev., June 1985, coll. Weir, Lawrence, Johnson (**ANIC**).

Australomimetus maculosus (Rainbow, 1904). **Australia: New South Wales:** 3 ♀, 1 ♂, 1 juvenile ♂ (4th instar) Lake Munmorah State Recreation Reserve, The Palms, 30 October 1987, coll. MR Gray (**AM**-KS17819); 1 ♀ Mt Colah (northern Sydney suburb), 23 Neridah Avenue, 29 November 2011, coll. MR Gray, HL Smith, GJ Anderson (**ZMH**-A0002038, GJA6532); 2 ♂ (♂ with less extensive carapace markings had spinneret developmental abnormalities) 0.6 km Batemans Bay, nests of *Ropalidia plebeiana*, 35°39'S 150°09'E, 23 May 2004, coll. J Kojima (**ZMH**-A0002027); 1 ♂ Tuglow, Kanangra-Boyd National Park, Tuglow Cave T1, 6 March 1993, coll. S Eberhard, det. MR Gray 1993 (**AM**-KS034965); 1 ♂, 1 juvenile ♂ (6th instar) Coolah Tops National Park, off Gemini Road Loop, sifting/beating in eucalypt forest with tree fern gully, 31°48'59"S 150°10'31"E, 1159 m elev., 12-13 April 2010, coll. MG Rix, D Harms (**ZMH**-A0002032; **ZMH**-A0002031); 1 juvenile ♂ (5th instar) Newcastle, Jesmond, 30 May 2014, coll. GJ Anderson (**ZMH**-A0002035, GJA8027). **Queensland:** 1 ♀ Far North Queensland, Dinden National Park, 30 September 2011, coll. GJ Anderson (**ZMH**-A0002036, GJA6383); 1 subadult ♀ (6th instar) Bulburin (Forestry Nursery) NW of Bundaberg, Rainforest Site 3A, foliage, 0016, 24°31'S 151°29'E, 580 m elev., March 1975, coll. M Gray, C Horseman (**AM**-KS0098); 1 juvenile ♀ (5th instar), 1 juvenile ♂ (4th instar) Far North Queensland, Julatten, Kingfisher Park, 29 December 2010, coll. GJ Anderson (**ZMH**-A0002047, GJA5841 ♀; **ZMH**-A0002048, GJA5852 ♂). **Tasmania:** 1 ♂ Flowery Gully Cave, FG201, 18 May 1989, coll. S Eberhard, J Jackson, det. MR Gray (**AM**-KS29547); 1 subadult ♂ (7th instar) Launceston, Cataract Gorge, 27 December 2012, coll. GJ Anderson (**ZMH**-A0002037, GJA7378); 1 juvenile ♀ (5th instar) Mt Field National Park, camping ground, on wall of toilet block, 42°41'05"S 146°42'57"E, 162 m elev., 1 January 2010, coll. MS Harvey, ME Blosfelds, F Harvey, E Harvey (**WAM**-T146229).

Australomimetus mendax Harms and Harvey, 2009. **Australia: Tasmania:** 2♂ Mt Wellington, Shoobridge Track, mixed eucalypt/rainforest (leaf litter & low vegetation), 42°54'30"S 147°14'53"E, 600 m elev., 8 October 2010, coll. D Harms, MG Rix (**ZMH**-A0002028); 4♂ W of Taylors Ridge, track from Hartz Mountains hut to Kermandie Plains, *Richea pandanifolia* beating, 43°13'01"S 146°46'56"E, 24 August 1973, coll. JL Hickman et al., det. D Harms, December 2014 (**AM**-KS107384); 1 subadult ♂ Mole Creek Karst National Park, track to King Solomons Cave (MC-119), mixed eucalypt forest with tree ferns, 41°33'07"S 146°14'55"E, 454 m elev., 18 October 2010, coll. D Harms, MG Rix (**ZMH**-A0002029).

Australomimetus mendicus (O Pickard-Cambridge, 1880). **New Zealand: Wellington:** 1♂ Stokes Valley, Raukawa Street, 41°11.2'S 174°58.8'E, 15 July 2013, coll. BM Fitzgerald (**ZMH**-A0002069).

Australomimetus sydneyensis Heimer, 1986. **Australia: New South Wales:** 3♂ Macquarie Pass National Park, off Clover Hill Road, sifting/beating in subtropical rainforest and hand-collecting at night, 34°34'05"S 150°39'25"E (WGS84), 828 m elev., 8 April 2010, coll. MG Rix, D Harms (**ZMH**-A0002025).

Australomimetus sp. nov. 'AU1'. **Australia: Victoria:** 1♂ Great Otway National Park, track to Triplet Falls, sifting/beating in temperate rainforest, 38°40'13"S 143°29'48"E (WGS84), 27 March 2010, coll. MG Rix, D Harms (**ZMH**-A0002026).

Australomimetus sp. nov. 'AU2'. **Australia: Queensland:** 1♂ 28°13'37"S 153°07'52"E, Plot # IQ-900-D, rainforest flight intercept trap, 920 m elev., 22 March—1 April 2007, coll. G Monteith, R Menendez (**QM**-S96312).

Australomimetus sp. nov. 'AU3'. **Australia: Queensland:** 1 subadult ♂ 28°08'53"S 153°08'13"E, Plot # IQ-300-A, rainforest flight intercept trap, 267 m elev., 21-31 March 2007, coll. G Monteith, R Menendez (**QM**-22182).

Australomimetus sp. nov. 'AU4'. **Australia: New South Wales:** 1♂ Dorrigo National Park, Rosewood Creek Circuit from Never Never Picnic Area, sifting/beating in subtropical rainforest, 30°21'42"S 152°47'55"E, 1092 m elev., 17 April 2010, coll. MG Rix, D Harms (**ZMH**-A0002030).

Australomimetus sp. nov. 'NC'. **New Caledonia:** 1♂ Aoupinie, Site # 03, by camp at night, 21°10'41"S 165°19'24"E (+-), 500 m elev. approx., 2-3 November 2001, coll. LJ Boutin (**WAM**-T66681).

Australomimetus sp. nov. 'NZ'. **New Zealand: Waikato:** 1♂ Hamilton, 37°44'17"S 175°14'49"E, 14 June 2014, coll. BN McQuillan (**ZMH**-A0002070); 1♂ Flagstaff, 37°44'06"S 175°14'24"E, 21 April 2010, coll. BN McQuillan (**ZMH**-A0002071).

Australomimetus sp. nov. 'NB'. **Papua New Guinea: New Britain:** 1♂ Camp 2, 14 May 2009, coll. I Agnarsson (**ZMH**-A0002023).

Australomimetus sp. nov. 'PNG'. **Papua New Guinea: Western Province:** 1♂ Muller Range (Camp 2), 05°40'S 142°18'E, 1425-1660 m elev., 4-14 September 2009, coll. I Agnarsson (**ZMH**-A0002024).

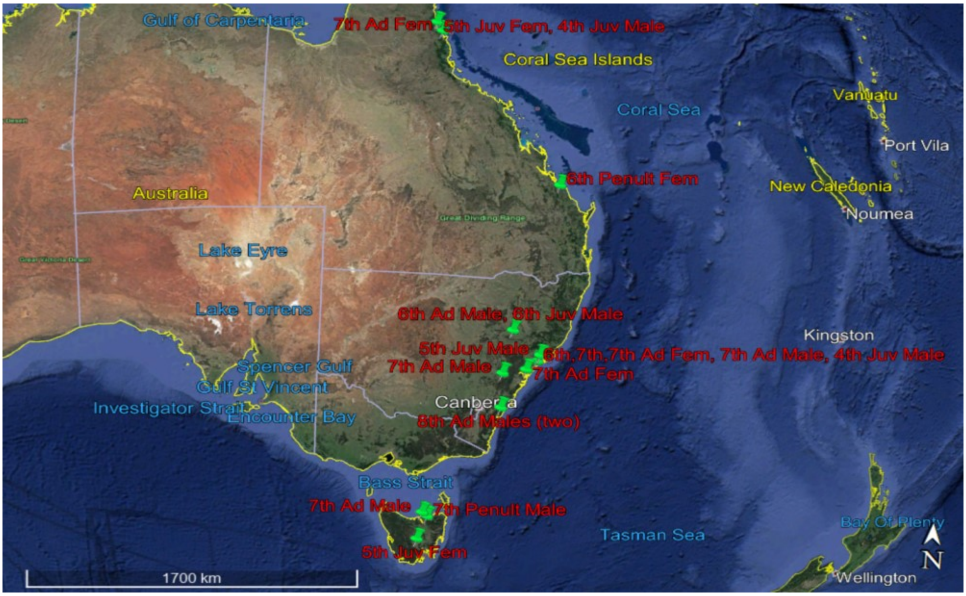

Sites of *A. maculosus* specimen collection indicated by green push-pins. Red labels adjacent to collection sites indicate stadium number, maturity, and sex of collected specimens. Ad, adult; Fem, female; Juv, juvenile; Penult, penultimate instar. Map made in Google Earth Pro version 7.3.2.5776; https://www.google.com/earth/versions/#earth-pro.

References (same numbering as in main paper)

19. Townley, M. A. & Harms, D. Comparative study of spinning field development in two species of araneophagic spiders (Araneae, Mimetidae, *Australomimetus*). *Evol. Syst.* **1,** 47–75; https://doi.org/10.3897/evolsyst.1.14765 (2017).
